# Targeting the AMP-Activated Protein Kinase for Cancer Prevention and Therapy

**DOI:** 10.3389/fonc.2013.00175

**Published:** 2013-07-15

**Authors:** InYoung Kim, Yu-Ying He

**Affiliations:** ^1^Pritzker School of Medicine, University of Chicago, Chicago, IL, USA; ^2^Department of Medicine, University of Chicago, Chicago, IL, USA

**Keywords:** AMPK, cancer prevention, DNA repair, proliferation, apoptosis, phytochemicals

## Abstract

Despite the advances in biomedical research and clinical applications, cancer remains a leading cause of death worldwide. Given the limitations of conventional chemotherapeutics, including serious toxicities and reduced quality of life for patients, the development of safe and efficacious alternatives with known mechanism of action is much needed. Prevention of cancer through dietary intervention may hold promise and has been investigated extensively in the recent years. AMP-activated protein kinase (AMPK) is an energy sensor that plays a key role in the regulation of protein and lipid metabolism in response to changes in fuel availability. When activated, AMPK promotes energy-producing catabolic pathways while inhibiting anabolic pathways, such as cell growth and proliferation – thereby antagonizing carcinogenesis. Other anti-cancer effects of AMPK may include promoting autophagy and DNA repair upon UVB damage. In the last decade, interest in AMPK has grown extensively as it emerged as an attractive target molecule for cancer prevention and treatment. Among the latest developments is the activation of AMPK by naturally occurring dietary constituents and plant products – termed phytochemicals. Owing to their efficacy and safety, phytochemicals are considered as an alternative to the conventional harmful chemotherapy. The rising popularity of using phytochemicals for cancer prevention and therapy is supported by a substantial progress in identifying the molecular pathways involved, including AMPK. In this article, we review the recent progress in this budding field that suggests AMPK as a new molecular target in the prevention and treatment of cancer by phytochemicals.

## Introduction

Despite the advances in biomedical research and clinical applications, cancer remains a leading cause of death worldwide. Given the limitations of conventional chemotherapeutics, including serious toxicities and reduced quality of life for patients, the development of safe and efficacious alternatives with known mechanism of action is much needed. In recent years, there has been increasing interest in the potential cancer chemopreventive properties of diet-derived agents, and many studies suggest that prevention of cancer through dietary intervention may hold promise. It is estimated that an average of 35% of human cancers (certain types up to 70%) can be attributed to diet (1), and epidemiological research has shown a link in the geographical distribution of cancer incidence to specific diet consumption. According to the World Health Organization report 2002, there are at least 2.7 million deaths globally per year, which are primarily attributable to low fruit and vegetable intake (2). This is not surprising, as the National Cancer Institute identified about 35 plant-based foods that possess anti-cancer benefits, including garlic, soybeans, ginger, onion, turmeric, tomatoes, and cruciferous vegetables (broccoli, cabbage, cauliflower, and Brussels sprouts). Furthermore, the chemopreventive efficacy of these diet constituents has been demonstrated *in vitro* and *in vivo*. The substantial anticarcinogenic and antimutagenic properties of these tested dietary agents can be attributed to the non-nutritive components of these foods, termed phytochemicals. There could be more than 100 different phytochemicals in just a single serving of vegetables (2), and they can be extracted for therapeutic purposes. Since phytochemicals have not been shown to have any known toxicities, they can be considered as an alternative to the conventional chemotherapy that may be harmful. A number of phytochemicals have been found to have notable efficacy in preclinical models of carcinogenesis, such as those of colorectum, breast, lung, and hematological origin. These include epigallocatechin gallate (EGCG) from tea, the flavonoids quercetin and genistein from onions and soya, respectively, curcumin in curry spice and resveratrol from red grapes (3). Chemopreventive phytochemicals can block the initiation or reverse the promotion of carcinogenesis and impede the progression of precancerous cells into malignant ones. A substantial progress has been made in identifying the signal transduction pathways involved in the antineoplastic effects of these substances, such as MAPK, MEK, PI3K, AP-1, COX-2, JNK, ERK, AP-1, and p53 (3–8). Elucidating the molecular mechanisms of phytochemicals fortifies the possibility in developing safe and effective preventative and therapeutic agents for cancer. In this review, we highlight (1) the role of the energy sensing enzyme AMP-activated protein kinase (AMPK) (9–11) in the prevention and treatment of cancer and (2) the recent developments in this budding field that suggest AMPK as a new molecular target in the prevention and treatment of cancer by phytochemicals.

## Function and Regulation of AMPK

AMP-activated protein kinase is a well-conserved energy sensor that plays a key role in the regulation of protein and lipid metabolism in response to changes in fuel availability (9– 11). AMPK exists as heterotrimeric complexes comprising a catalytic α-subunit and regulatory β- and γ-subunits. In mammals, each subunit occurs as multiple isoforms encoded by multiple genes that can be assembled to form up to 12 heterotrimeric combinations (12). Although AMPK is traditionally thought to play a major role in the regulation of cellular metabolism, it is now widely recognized to have antineoplastic efficacy and as a target for chemotherapy. A key characteristic of tumor cells is their ability to rapidly grow and divide, thus requiring a tremendous demand for energy. An extensive body of evidence has demonstrated that AMPK inhibits essentially all anabolic pathways that promote cell growth, such as synthesis of fatty acid, phospholipid, protein, and ribosomal RNA synthesis (12, 13). Thus, it is not surprising that AMPK antagonizes cancer cell growth. The first hint that AMPK may be linked to cancer was provided by the finding that liver kinase B1 (LKB1), a known tumor suppressor, acted as an upstream kinase of AMPK (14, 15). LKB1 is mutated in the inherited cancer disorder Peutz–Jeghers syndrome and in many lung and cervical cancers, suggesting that AMPK could play a role in tumor suppression (16, 17).

### Activators of AMPK

AMP-activated protein kinase can be activated by various types of metabolic stress that lead to ATP depletion, such as conditions of low nutrient supply or prolonged exercise, or via an increase in intracellular Ca^2+^ concentration (9). The upstream kinases, LKB1 and calcium/calmodulin-dependent protein kinase kinase-β (CaMKKβ) activate AMPK by phosphorylating Thr172 in the activation loop of the catalytic α-subunit (18–20). The finding that CaMKKβ can also activate AMPK, independently of LKB1, broadened the potential for AMPK to be used for therapy in cancers that have mutant LKB1 and thus low AMPK activation. Loss of function of the tumor suppressor LKB1 occurs in 30–50% of lung adenocarcinomas. Memmott et al. (21) found that lipid-based AKT inhibitors, phosphatidylinositol ether lipid analogs (PIA), activate AMPK independently of LKB1 in LKB1-mutant non-small cell lung cancer (NSCLC) cell lines. The more well-known activators of AMPK include several pharmacological agents that stimulate the LKB1 pathway. Metformin, the most widely prescribed Type-2 diabetes drug for more than 30 years, has been shown to activate AMPK in an LKB1-dependent manner (22, 23). Metformin mimics an energetic stress because it inhibits the mitochondrial complex I in hepatocytes and cancer cells which leads to the decrease in intracellular ATP and an increase in glycolysis and lactate production (24, 25). Consistent with this, diabetic patients treated with metformin had a lower incidence of cancer compared to those on other medications (26). In light of this, other retrospective studies have been performed, one of which showed that breast cancer patients on metformin for diabetes responded significantly better to chemotherapy than other diabetic patients not on metformin and non-diabetic patients (27). Since then, several studies have shown that metformin exerts antineoplastic effects in other cancer cells and animal models. Phenformin, a biguanide more potent than metformin, and A-769662, a direct AMPK activator developed by Abbott also delayed tumorigenesis in a mouse cancer model (28). 5-Amino-1-β-Dffff-ribofuranosyl-imidazole-4-carboxamide, or AICAR, is an analog of AMP and widely used to activate AMPK in experiments. Interestingly, AMPK is also activated by ionizing radiation (IR) in lung, prostate, and breast cancer cells, independent of LKB1. This suggests that AMPK may play a role as a target for radiosensitization of human cancer cells (29). Since the discovery of antineoplastic effects of AMPK, the number of patents describing potential AMPK activators has grown rapidly (30, 31). Among the most recent developments is the activation of AMPK by naturally occurring dietary constituents and plant products, to be reviewed in the latter part of this article.

### Downstream targets of AMPK

**Figure 1:**
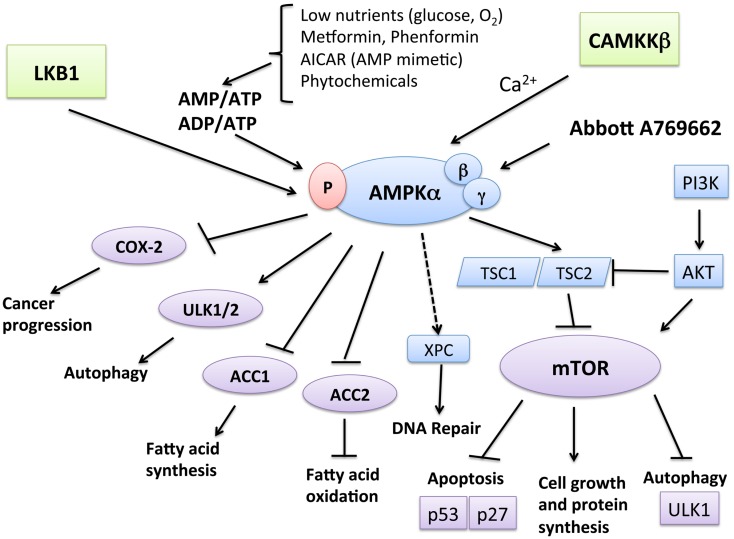
**Function and regulation of AMPK leading to tumor suppression**. AMPK is activated when AMP/ATP or ADP/ATP ratios in the cells rise due to various physiological stresses, such as hypoglycemia and hypoxemia, leading to the activation of LKB1. Metformin and phenformin can also mimic these stressors and lead to AMPK activation in a LKB1-dependent manner. CaMKKβ activates AMPK in response to calcium increase. Catabolic pathways, such as fatty acid oxidation, are activated by AMPK. For example, AMPK phosphorylation leads to the inactivation of acetyl CoA carboxylase (ACC2). On the other hand, AMPK inhibits anabolic pathways, such as fatty acid synthesis, mediated by ACC1. One of the most well-known pathways of AMPK is through the TSC1/TSC2 complex, leading to the downregulation of mTOR, which can also can be activated downstream of the PI3K-AKT and Ras-Raf-MEK-ERK signaling pathways. The mTOR pathway suppresses apoptosis via its effect on the tumor suppressors p53 and p27 and inhibits autophagy by suppressing UNC-51-like kinase 1 (ULK1) and ULK2. AMPK downregulates these effects of mTOR, thus leading to increased apoptosis and autophagy-mediated cell death. Independent of mTOR, AMPK phosphorylates and activates ULK1 and ULK2, thus triggering autophagy. Furthermore, AMPK has been shown to downregulate the expression of cyclooxygenase (COX)-2, which contributes to the pathophysiological progression of certain human cancers and inflammatory disorders. AMPK is necessary for the expression of xeroderma pigmentosum C (XPC) to promote DNA repair following UV damage.

One of the major growth regulatory pathways controlled by LKB1–AMPK is the mammalian target of rapamycin (mTOR) pathway (Figure 1). The mTOR pathway controls various biological processes that are important for normal functioning of the cell via cell-cycle progression, survival, migration, transcription, translation, and metabolism. AMPK is linked with the phosphatidylinositol 3-kinase (PI3K)/phosphatase and tensin homolog (PTEN)/protein kinase B (AKT) pathway and mitogen-activated protein kinase (MAPK)/extracellular signal-regulated kinases (ERK) cascades – all known for being frequently dysregulated in cancer. mTOR can be activated downstream of the PI3K-AKT and Ras-Raf-MEK-ERK signaling pathways (32), which converge on TSC1-TSC2 (33). Moreover, AMPK has been shown to be necessary for cell-cycle arrest at the G1 phase during limited nutrient supply, via phosphorylation of the tumor suppressors p53 (34, 35) and p27 (36). The mTOR complex also controls AMPK-mediated autophagy (37), a cellular process in which the cell breaks down its own organelles and cytosolic components to ensure sufficient metabolites during starvation states. Under certain circumstances, autophagic cells may engage a specific mode of cell death called type II cell death or autophagic cell death (ACD). A number of studies have revealed the role of AMPK in ACD of cancer cells (38, 39). The most upstream components of the autophagy pathway include Atg1 (ULK1 in mammals) and its regulatory subunits Atg13 and Atg17 (40). The mTOR/Raptor pathway is thought to suppress ULK1 and ULK2 and their regulatory subunits (41), while AMPK phosphorylates ULK1, leading to autophagy-mediated cancer cell death. Furthermore, AMPK has been shown to downregulate the expression of cyclooxygenase (COX)-2, which contributes to the pathophysiological progression of certain human cancers and inflammatory disorders (42). AMPK is required for the expression of xeroderma pigmentosum C (XPC) to promote DNA repair following UV damage (43).

## Role of AMPK in Cancer Cell Lines and Animal Models of Cancer

Since the AMPK cascade has emerged as an important pathway implicated in cancer control, many discoveries have been made in the past decade revealing robust anti-cancer effects of AMPK *in vitro* and *in vivo*, including animal cancer models of breast, lung, colorectum, skin, and hematological malignancies. We review the role of AMPK in major cancers, as outlined below.

### Breast cancer

Observations that diabetics treated with biguanide drugs have a reduced risk of developing cancer have prompted an enthusiasm for these agents as anti-cancer therapies. Metformin, which activates AMPK, was shown to inhibit cell proliferation and induce apoptosis in the triple-negative breast cancer cell line, as well as the estrogen receptor (ER) α-positive and the human epidermal receptor (HER) 2-positive cell lines. Moreover, synergistic inhibition of the G1 phase of the cell cycle was demonstrated by the combination treatment of metformin and chemotherapeutic agents carboplatin, paclitaxel, and doxorubicin (44). Moreover, a novel small molecule AMPK activator, OSU-53 derived from inactive peroxisome proliferator-activated receptor gamma (PPARγ), was reported to inhibit the proliferation of the triple-negative breast cancer, a disease for which there are limited therapeutic options (45). Phenformin, a stronger biguanide than metformin and also a direct activator of AMPK, was demonstrated to be effective in the prevention and treatment of ER-positive and receptor triple-negative xenografts in immunocompromised mice (46). In another study, AICAR and phenformin elicited clear anti-proliferative effects in ER-positive, ER-negative, and triple-negative breast cancer cell lines (47).

### Lung cancer

AMP-activated protein kinase is considered a potential prognostic and therapeutic target for lung cancer. In tumors from patients with resected non-small cell lung cancer (NSCLC), the expression of proteins in the AMPK pathway, including pLKB1, AMPK, *p*-Acetyl-CoA, pTSC2, was inversely correlated with NSCLC recurrence (48). Consistent with this, another study showed a significant association between high phosphorylated AMPK (pAMPK) expression levels with increased overall survival and recurrence-free survival in patients with NSCLC, especially those with adenocarcinoma (49). Never smokers also showed significantly higher levels of pAMPK compared to former and current smokers. In NSCLC cells *in vitro*, LKB1/AMPK signaling was shown to negatively regulate mTOR activity and contribute to cell growth inhibition in response to 2-deoxyglucose (2-DG), which mimics energy stress (50). Moreover, metformin treatment led to increased apoptosis in human lung cancer cell lines (A549 and NCI-H1299) and significantly inhibited cell proliferation in a dose- and time-dependent manner, which was confirmed by results from A549 tumor xenografts in nude mice (51). Similarly, A/J mice treated with oral metformin after exposure to the tobacco carcinogen 4-(methylnitrosamino)-1-(3-pyridyl)-1-butanone (NNK) showed a 72% reduction in tumor burden compared to the control mice, which correlated with decreased cellular proliferation and marked inhibition of mTOR in the tumors (52).

### Hematological cancers

AMP-activated protein kinase has been shown to inhibit cancer cell growth in various hematological cancers. In acute lymphoblastic leukemia (ALL) cell lines, AICAR induced dose- and time-dependent cell growth inhibition (53), leading to increased AKT phosphorylation and decreased mTOR phosphorylation (54). When used in combination with methotrexate or pemetrexed, AMPK showed a synergistic cytotoxic effect and cell growth inhibition (53). Sengupta et al. (55) reported that the apoptotic effect of AMPK was mediated by activation of the p38 MAPK pathway, increased expression of cell-cycle inhibitory proteins p27 and p53, and the downstream effects of the mTOR pathway. In B-cell chronic lymphocytic leukemia (B-CLL) cells, AMPK-induced apoptosis in a p53-independent manner (56). In mantle cell lymphoma (MCL), a clinically aggressive B-cell non-Hodgkin lymphoma characterized by the t(11;14)(q13;q32) and overexpression of cyclin D1, stimulation of the AMPK kinase activity using AICAR inhibited phosphorylation of critical downstream effectors of mTOR signaling, such as 4E-BP1 and ribosomal protein s6 (rps6) (57). In BCR-ABL-expressing chronic myeloid leukemia (CML) precursors and ALL cells that are positive for the Philadelphia chromosome (Ph+), metformin, and AICAR suppressed the mTOR pathway and cell growth (58). Lastly, induction of the LKB1/AMPK tumor suppressor pathway demonstrates a strong potential for the treatment of acute myeloid leukemia (AML). Green et al. showed that the LKB1/AMPK/TSC tumor suppressor axis could lead to a specific inhibition of the mammalian target of rapamycin (mTOR) catalytic activity, inducing 4E-BP1 dephosphorylation, which inhibits the initiation step of mRNA translation. Metformin consequently reduced the recruitment of mRNA molecules encoding oncogenic proteins to the polysomes, resulting in a strong anti-leukemic activity against primary AML cells while sparing normal hematopoiesis *ex vivo* and significantly reducing the growth of AML cells in nude mice (59).

### Skin cancer

The role of AMPK in UVB-induced skin cancer is still only beginning to be understood and depends on the type of skin cancer. AMPK activators phenformin and AICAR were shown to inhibit the cell growth of both BRAF-mutant or NRAS-mutant melanoma cell, due to cell-cycle arrest in either the G0/G1 or the S phase, associated with an increased expression of the p21 cell-cycle inhibitor (60). However, another study revealed that BRAF-mutant melanoma cells are resistant to metformin *in vitro*, while metformin accelerates their growth *in vivo*. Surprisingly, metformin inhibited tumor growth when vascular endothelial growth factor (VEGF) signaling was inhibited. Thus, VEGF inhibitors and metformin synergized to suppress the growth of BRAF-mutant tumors (61). The role of AMPK in basal cell carcinoma remains unclear. In a study by Byekova et al. (62) LKB1 and pAMPK expression was shown to be upregulated in UVB-induced murine BCC and in human skin tumor keratinocytes. Paradoxically, persistent mTOR activation was also observed. Metformin was effective in activating the LKB1/AMPK pathway only in HaCaT keratinocytes but not in human carcinoma A431 cells, suggesting a complex regulatory mechanism for the persistent mTOR activation in murine BCCs. In contrast, Zhang and Bowden reported that UVB irradiation, a strong carcinogen for non-melanoma skin cancer, reduced activation of AMPK and LKB1, leading to increased Cox-2 mRNA stability, which may contribute to cancer development (63). Furthermore, our recent studies showed that the AMPK pathway is down-regulated in human and mouse squamous cell carcinomas and that its activators AICAR and metformin increased the expression of the DNA repair protein xeroderma pigmentosum C (XPC) and UVB-induced DNA repair in mouse skin and in normal human epidermal keratinocytes (43). Furthermore, in UVB-damaged tumor-bearing mice, both topical and systemic metformin prevented the formation of new tumors and suppressed growth of established tumors, demonstrating that AMPK acts as a tumor suppressor in the skin by promoting DNA repair and controlling cell proliferation (43).

## Activation of AMPK by Phytochemicals

It was not until about 5 years ago that AMPK began to be recognized as a target for various phytochemicals. In this section, we review the studies that have demonstrated the role of AMPK in the chemopreventive effects of phytochemicals (Table 1), the majority of which have been reported in the last few years.

**Table 1:** **Chemopreventive/chemotherapeutic phytochemicals that activate AMPK**.

Phytochemical	Effect	Reference
**CURCUMIN**		
Curcumin (diferuloylmethane), from turmeric (*Curcuma longa* L.)	Activates AMPK to induce cell death in CaOV3 ovarian cancer cells in a p38 MAPK-dependent manner	(67)
	Stimulates AMPK, resulting in downregulation of PPARγ in 3T3-L1 adipocytes and in COX-2 in MCF-7 breast cancer cells, inhibiting differentiation and growth	(65)
	Inhibits mTOR, independent of AMPK	(64)
	Downregulates COX-2 and pAKT in an AMPK-dependent manner, leading to apoptosis of H29 colon cancer cells	(66)
**GRAPE POLYPHENOLS**		
Resveratrol	Induces apoptosis in chemoresistant HT-29 colon cancer cells via modulation of AMPK signaling pathway	(74)
	Activates AMPK and suppresses LPS-induced NF-κB-dependent COX-2 activation in RAW 264.7 macrophage cells	(75)
	Promotes autophagy-mediated cell death in chronic myelogenous leukemia cells in an AMPK-dependent manner	(76)
	3,4-DMS, a methylated resveratrol derivative, induced autophagy in endothelial cells through activation of AMPK and the downstream inhibition of mTOR signaling pathway	(80)
	Activates AMPK via SIRT1 in both ER-positive and ER-negative breast cancer cells, leading to inhibition of 4E-BP1 signaling and mRNA translation via mTOR	(105)
	Enhances prostate cancer cell response to ionizing radiation by modulation of AMPK	(78)
	Inhibits AKT/mTOR signaling via AMPK and potentiates the effects of gefitinib in breast cancer	(77)
	Enhances anti-tumor effects of temozolomide in glioblastoma via ROS-dependent AMPK-TSC-mTOR signaling pathway	(79)
**FLAVONOIDS**		
Apigenin	Induces AMPK and autophagy, inhibiting mTOR, and further inducing autophagy in both HaCaT cell line and primary normal human epidermal keratinocytes. This effect was independent of AKT and LKB1 but dependent on CaMMKβ	(90)
Anthocyanin	Activates AMPK, leading to a reduction in mTOR phosphorylation and inhibition of HT-29 colon cancer cell growth	(106)
Fisetin	Activates AMPK to induce apoptosis in multiple myeloma cells	(85)
	Inhibits PI3K/Akt and mTOR and activates AMPK in non-small cell lung cancer	(86)
	Induces autophagy-mediated cell death by suppressing mTOR in prostate cancer cells	(87)
Quercetin	Induces apoptosis via AMPK activation and p53 in HT-29 colon cancer cells	(88)
	Suppresses cell viability via AMPK-induced Hsp70 and EGFR downregulation	(89)
Baicalein	Induces apoptosis and AMPK in human tumor cells	(107)
Luteolin	Induces cell death in HepG2 cells and reduces tumor volume in a tumor xenograft model	(91)
Hispidulin	Activates AMPK and inhibits downstream mTOR, which induces apoptosis in glioblastoma multiforme cells by p53 and p21 induction	(108)
Genistein	Decreases reactive oxygen species levels and induces antioxidant enzymes manganese superoxide dismutase and catalase in a AMPK and PTEN-dependent manner in prostate cancer cells	(82)
	Potentiates arsenic trioxide-induced apoptosis in human leukemia cells by activation of AMPK	(83)
Deguelin	Activates AMPK and inhibits UVB-induced tumorigenesis in the SKh-1 hairless mouse model	(92)
Tephrosin (plant rotenoid)	Enhances cytotoxicity of anti-cancer agent via ATP depletion and reducing autophagy by activation of AMPK and inactivation of mTOR expression	(109)
Chrysin	Leads to cell growth inhibition and apoptosis in lung cancer cells via activation of AMPK and inhibition of AKT/mTOR	(94)
Celastrol	Suppresses breast cancer MCF-7 cell viability via the AMP-activated protein kinase (AMPK)-induced p53-polo like kinase 2 (PLK-2) pathway	(93)
**GREEN TEA POLYPHENOLS**		
Epigallocatechin gallate (EGCG)	EGCG analogs activate AMPK, leading in inhibition of cell proliferation, up-regulation of the cyclin-dependent kinase inhibitor p21, downregulation of the mTOR pathway, and suppression of stem cell population in human breast cancer cells	(96)
	Enhances 5-fluorouracil-induced cell growth inhibition of hepatocellular carcinoma cells, associated with AMPK hyperactivation and COX-2 inhibition	(98)
	Activates AMPK in the liver and prevents diethylnitrosamine-induced liver tumorigenesis in obese and diabetic mice	(97)
	Induces apoptosis in HT-29 colon cancer cells via the AMPK/COX-2 pathway	(95)
Catechin	Induces apoptosis in colon cancer cells by attenuation of H_2_O_2_-stimulated COX-2 expression via AMPK	(110)
**OTHER CATEGORY**		
*p*-HPEA-EDA, phenolic compound of virgin olive oil	Activates AMPK to suppress COX-2 and inhibit cell survival in HT-29 colon cancer cells	(102)
24-Hydroxyursolic acid from persimmon	Activates AMPK and induces apoptosis in HT-29 colon cancer cells; Also block EGF-induced ERKs phosphorylation and inhibits AP-1 activity and cell transformation	(101)
Capsaicin	Induces apoptosis in HT-29 colon cancer cells, which correlated with AMPK activation in capsaicin-treated colon cancer cells	(103)
Berberine	Inhibits colon cancer migration via AMPK activation-mediated downregulation of integrin b1 signaling	(100)
	Berberine-induced AMPK activation inhibits the metastatic potential of tumor cells through a reduction in the activity of the ERK signaling pathway and COX-2 protein levels	(99)

### Curcumin

**Figure 2:**
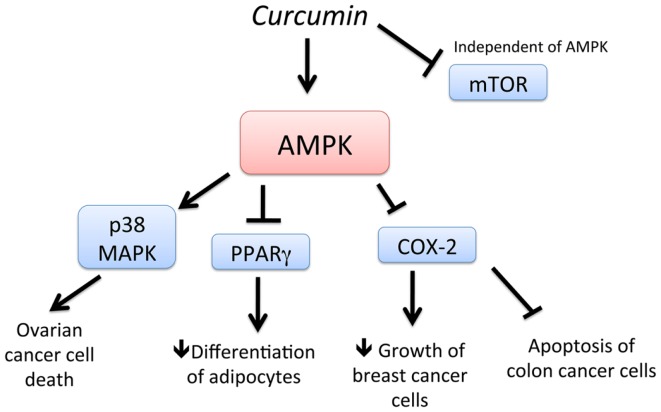
**Schematic representation of AMPK-dependent anti-cancer effects of curcumin**. Curcumin activates AMPK and increases cell death of ovarian cancer cells, in a p38 MAPK-dependent manner. Activation of AMPK by curcumin also leads to downregulation of PPAR g and COX-2, leading to decreased differentiation of adipocytes and delayed growth of breast cancer cells, respectively. Downregulation of COX-2 also leads to apoptosis of colon cancer cells. In addition, curcumin downregulates mTOR, independent of AMPK.

Curcumin (diferuloylmethane), a yellow pigment present in the rhizome of turmeric (*Curcuma longa* L.), is one of most extensively investigated phytochemicals in the field of chemoprevention and is used in early clinical trials as a novel anti-cancer agent. Curcumin has been shown to suppress tumor progression in various animal models of cancer (64–67). Recently, AMPK was found to be a new molecular target of curcumin (Figure 2). Pan et al. (67) showed that activation of AMPK by curcumin has shown to be responsible for the cytotoxic effects of curcumin ovarian cancer cells. In another study, stimulation of AMPK by curcumin resulted in the downregulation of PPAR (peroxisome proliferator-activated receptor)-g in 3T3-L1 adipocytes and a decrease in COX-2 in MCF-7 cells (65). Application of a synthetic AMPK activator also supported the evidence that AMPK acts as an upstream signal of PPARγ in 3T3-L1 adipocytes. In cancer cells, AMPK was found to act as a regulator of ERK1/2, p38, and COX-2. Thus, activation of AMPK by curcumin and its downstream targets such as PPAR-g, MAP kinases, and COX-2 is important in regulating adipocytes and cancer cells (66). Consistent with this study, curcumin activated AMPK to induce apoptosis and limit proliferation of colon cancer cells via the inhibition of AKT and COX-2 (66). Thus, curcumin is a potent stimulator of AMPK leading to chemoprevention.

### Resveratrol

**Figure 3:**
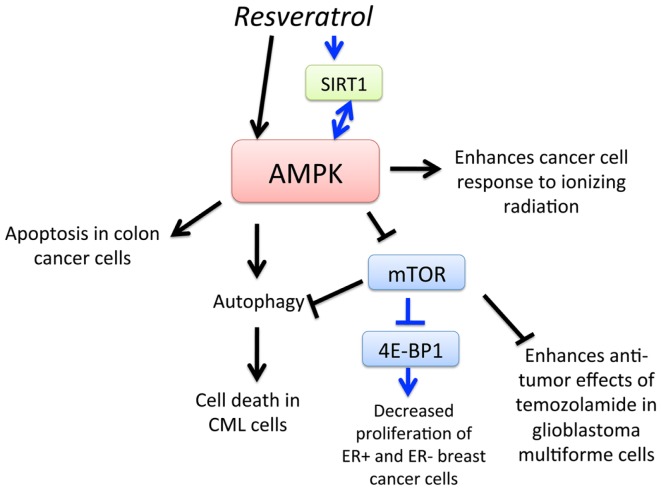
**Schematic representation of AMPK-dependent anti-cancer effects of resveratrol**. Activation of AMPK by resveratrol leads to apoptosis of colon cancer cells, enhancement of cancer cell response to ionizing radiation, and mTOR-dependent and – independent autophagy, leading to cell death in chronic myelogenous leukemia (CML) cells. Resveratrol also activates SIRT1, which leads to AMPK activation, leading to downregulation of mTOR and inhibition of 4E-BP1 and decreased proliferation of estrogen receptor-positive (ER+) and ER-negative breast cancer cells. Furthermore, activation of AMPK and subsequent downregulation of mTOR increases sensitivity of glioblastoma multiforme (GBM) cells to temozolomide.

Resveratrol (3,4′,5-trihydroxy-trans-stilbene) is a type of natural phenol that is present in grapes and a key antioxidant ingredient in red wine. The consumption of red wine has been correlated with the reduction of mortality rates from cardiovascular diseases and certain cancers. Moreover, anti-cancer, anti-inflammatory, blood sugar-lowering, and other beneficial cardiovascular effects of resveratrol have been reported in animal models and human clinical trials (68–71). One of the earliest findings of its anti-cancer effects was in 1997, in which topical resveratrol applications prevented skin cancer development in mice treated with a carcinogen (72). There have since been many studies demonstrating the anti-cancer activity of resveratrol in animal models (73). AMPK is now a recognized target of resveratrol that mediates its anti-cancer effects (Figure 3). In 2007, Hwang et al. (74) showed that resveratrol activates AMPK and induces apoptosis of chemoresistant HT-29 colon cancer cells and identified that reactive oxygen species (ROS) acted as an upstream regulator of AMPK. Resveratrol was also found to activate AMPK, leading to the suppression of NF-κB-dependent COX-2, a pathway implicated in cancer development (75). Moreover, resveratrol-induced autophagy-mediated cell death in imatinib-sensitive and – resistant CML cells. AMPK knockdown or mTOR overexpression impaired resveratrol-induced autophagy, suggesting that AMPK activation and mTOR inhibition is important for autophagy-mediated cancer cell death (76). Resveratrol also increased sensitivity to standard chemotherapies, thus reducing the required dosage of potentially toxic substances in prostate cancer, glioblastoma, and breast cancer cells (77–79). A series of screening resveratrol methylated derivatives was performed, and trans-3,4-dimethoxystilbene (3,4-DMS) was found to effectively inhibit endothelial cell proliferation, migration, tube formation, and endogenous neovascularization. Moreover, 3,4-DMS induced autophagy in endothelial cells through AMPK activation and downstream inhibition of the mTOR signaling pathway (80). As outlined in this section, evidence for the chemopreventive potential of resveratrol is growing, as the various molecular mechanisms of its action via AMPK is being elucidated.

### Flavonoids and related compounds

**Figure 4:**
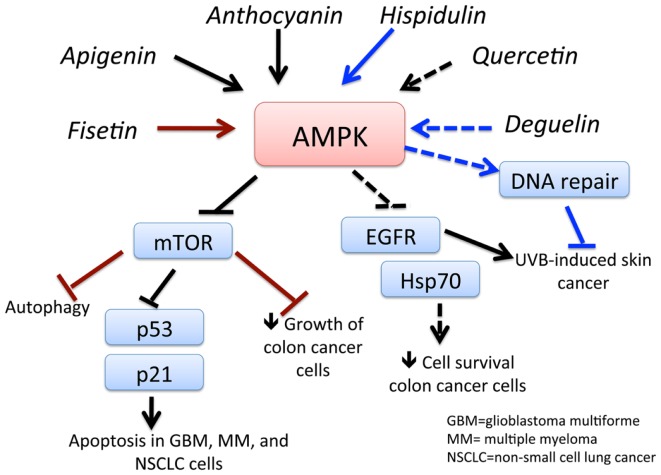
**Schematic representation of AMPK-dependent anti-cancer effects of flavonoids and related compounds**. This figure shows the major flavonoids and their AMPK-dependent effects on inhibition of cancer growth. Apigenin and anthocyanin activates AMPK, which inhibits mTOR signaling, leading to apoptosis of GBM, multiple myeloma (MM), and non-small cell lung cancer (NSCLC) cells. Activation of AMPK by fisetin and hispidulin also inhibits mTOR, resulting in autophagy-dependent cancer cell death and decreased growth of colon cancer cells, respectively. Quercetin inhibits survival of colon cancer cells by downregulation of epidermal growth factor receptor (EGFR) signaling and heat shock protein (hsp)70 expression. Activation of AMPK deguelin leads to UVB-induced tumorigenesis in a non-melanoma skin cancer mouse model. See text for effects of other flavonoids and related compounds.

Flavonoids can be found in many different sources, including soy, berries, tea, wine, beer, chocolate, many vegetables, and most fruits. While there are several 1000 types, they can be categorized as flavones (quercetin, fisetin, luteolin), isoflavonoids (genistein, deguelin), and neoflavonoids (81). Epidemiological evidence reveals a lower incidence of prostate cancer in Asian countries, where soy products are more frequently consumed than in Western countries. The chemopreventive effects of a soy isoflavonoid genistein can be attributed to its ability to activate AMPK (Figure 4) and PTEN, leading to the induction of the antioxidant enzymes manganese superoxide dismutase and catalase (82). In another study, genistein was demonstrated to potentiate arsenic trioxide-induced apoptosis in human leukemia cells by activation of AMPK (83). Moreover, anthocyanin – belonging to the family of flavones that occurs in all tissues of higher plants, including leaves, stems, roots, flowers, and fruits – is shown to be a powerful activator of AMPK. Lee and Park (84) demonstrated that anthocyanin activates AMPK, leading to a reduction in mTOR phosphorylation and ultimately inhibiting cancer cell growth. Fisetin, a flavonoid, activates AMPK to induce apoptosis in multiple myeloma cells (85), inhibits PI3K/AKT and mTOR and activates AMPK in non-small cell lung cancer (86), and induces autophagy-mediated cell death by activating AMPK and suppressing mTOR in prostate cancer cells (87). Quercetin induces apoptosis via AMPK activation and p53 in HT-29 colon cancer cells (88) and suppresses cell viability via AMPK-induced Hsp70 and EGFR downregulation (89). Apigenin induces AMPK, inhibiting mTOR and further inducing autophagy in both HaCaT cell line and primary normal human epidermal keratinocytes. This effect was independent of AKT and LKB1 but dependent on CaMMKβ (90). Luteolin showed its anti-tumor effects in an *in vivo* tumor model, in which its activation of AMPK reduced tumor volume in a tumor xenograft model (91). Deguelin, a plant-derived rotenoid with cancer chemopreventive activity, was shown to inhibit UVB-induced skin carcinogenesis with the SKh-1 hairless mouse model. Topically applied deguelin significantly inhibited the multiplicity of UVB-induced skin tumors by activating AMPK (92). Celastrol, another antioxidant flavonoid, suppressed the viability of breast cancer MCF-7 cells in an AMPK-dependent fashion. Celastrol also induced an increase in ROS levels, leading to AMPK phosphorylation and increased the pro-apoptotic p53 in an AMPK-dependent manner (93). Chrysin, a naturally occurring flavone chemically extracted from the passion flowers *Passiflora caerulea* and *Passiflora incarnata*, leads to growth inhibition and apoptosis of lung cancer cells via AMPK activation and inhibition of AKT/mTOR (94). Thus, flavonoids and related compounds act as direct activators of AMPK and demonstrate their promising potential to be used as chemotherapeutic agents.

### Epigallocatechin gallate

**Figure 5:**
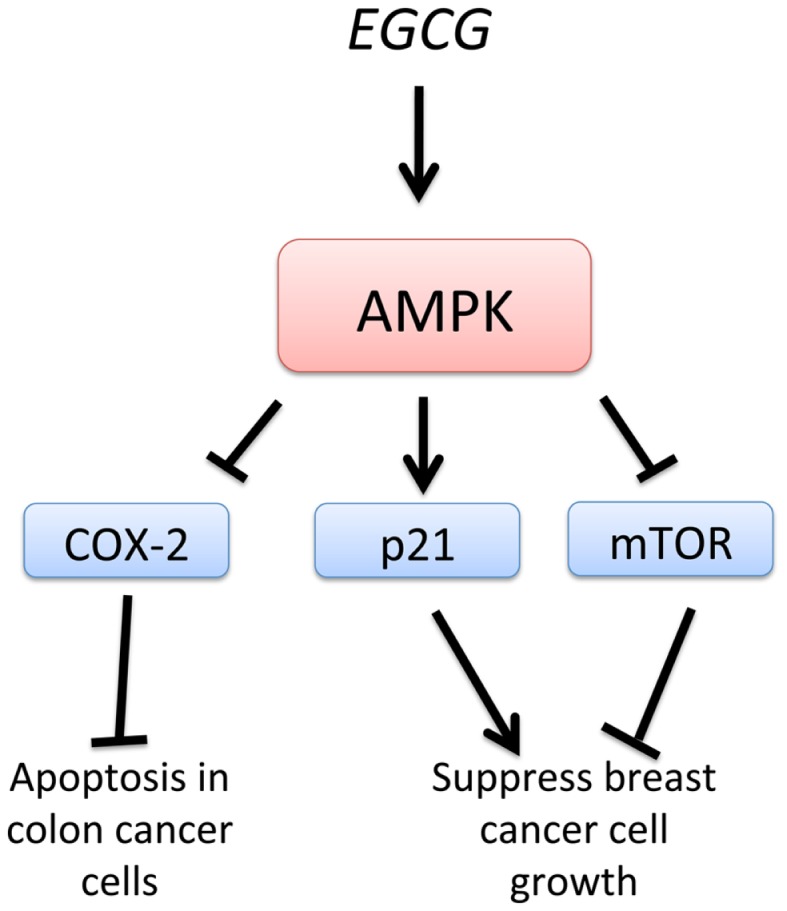
**Schematic representation of AMPK-dependent anti-cancer effects of EGCG**. Epigallocatechin-3-gallate, EGCG, stimulates AMPK, leading to suppression of breast cancer cell growth by inhibition of mTOR and activation of p21. Inhibition of COX-2 by EGCG-induced AMPK activation leads to apoptosis in colon cancer cells.

Epigallocatechin-3-gallate, EGCG, a green tea-derived polyphenol, has been shown to suppress cancer cell proliferation and interfere with the several signaling pathways and induce apoptosis. EGCG treatment of HT-29 colon cancer cells resulted in a strong activation of AMPK and an inhibition of COX-2 expression (Figure 5). Treatment with an AMPK inhibitor completely abolished the inhibition of COX-2 by EGCG. Also, AMPK activation was accompanied by a reduction of VEGF and glucose transporter, Glut-1 in EGCG-treated cancer cells. These findings support the regulatory role of AMPK in COX-2 expression in EGCG-treated cancer cells (95). Moreover, analogs of EGCG have been synthesized and found to be more potent AMPK activators than metformin and EGCG. Activation of AMPK by these EGCG analogs resulted in the inhibition of cell proliferation, up-regulation of the cyclin-dependent kinase inhibitor p21, downregulation of the mTOR pathway, and suppression of stem cell population in human breast cancer cells. This study suggests that specific and more potent AMPK activators can be derived from natural and synthetic sources and be used for chemotherapy (96). In a model of diethylnitrosamine-induced liver tumorigenesis in obese and diabetic mice, EGCG improved liver steatosis and activated AMPK in the liver, suggesting that EGCG may prevent obesity-related liver tumorigenesis (97). EGCG, therefore, may be useful in the chemoprevention of liver tumorigenesis in obese individuals by the activation of AMPK, consistent with the reported effects of metformin. EGCG is known to play a critical role in growth inhibition and apoptosis in hepatocellular carcinoma cell lines. Furthermore, EGCG was shown to enhance the anti-tumor activity of 5-fluorouracil (5-FU), one of the most commonly used chemotherapeutic drugs, suggesting that EGCG may be used as an adjunct therapy for the treatment of advanced-stage liver cancer (98).

### Phytochemicals belonging to other category

Berberine, a traditional plant alkaloid used in Ayurvedic and Chinese medicine for its antimicrobial and antiprotozoal properties, strongly increased AMPK phosphorylation via ROS production, leading to inhibition of tumor cell adhesion, tumor invasion, and the expression of epithelial to mesenchymal transition (EMT)-related genes. Furthermore, berberine inhibited the metastatic potential of melanoma cells through a decrease in ERK activity and protein levels of cyclooxygenase-2 (COX-2) by a berberine-induced AMPK activation (99). In another study, berberine was shown to inhibit migration of colon cancer cells in an AMPK-dependent manner (100). Furthermore, 24-hydroxyursolic acid from persimmon, capsaicin, and p-HPEA-EDA, a phenolic compound of virgin olive oil, activate AMPK and inhibit cell survival in HT-29 colon cancer cells (100–103).

## Conclusion and Future Considerations

In summary, the use of phytochemicals derived from dietary agents holds promise in the prevention and treatment of cancer, and AMPK is one of the major pathways activated by many phytochemicals. Not only do phytochemicals activate AMPK to increase cancer cell apoptosis and inhibit cell proliferation, but they have also been shown to be effective in reducing the toxicity associated with standard chemotherapy by increasing the sensitivity of cancer cells to drugs. While the majority of the studies on AMPK that we reported in this review demonstrate that it is an antineoplastic agent, some evidence suggests the role of AMPK in promoting cancer. This warrants further investigation. As AMPK exists as different heterotrimeric compound consisting of various isoforms, it is conceivable that different contexts of stimulation may lead to disparate consequences. There are other phytochemicals that have been reported to activate AMPK, such as Honokiol (104), that have been shown to have consequences other than cancer prevention. Thus, it seems that AMPK can be used not only as a chemotherapeutic agent but also to protect from injury, diabetes, or inflammatory diseases.

## Conflict of Interest Statement

The authors declare that the research was conducted in the absence of any commercial or financial relationships that could be construed as a potential conflict of interest.
